# Physicochemical Properties Influencing Presence of *Burkholderia pseudomallei* in Soil from Small Ruminant Farms in Peninsular Malaysia

**DOI:** 10.1371/journal.pone.0162348

**Published:** 2016-09-16

**Authors:** Hassan Ismail Musa, Latiffah Hassan, Zulkifli Hj. Shamsuddin, Chandrawathani Panchadcharam, Zunita Zakaria, Saleha Abdul Aziz

**Affiliations:** 1 Faculty of Veterinary Medicine, Universiti Putra Malaysia, Serdang, Selangor, Malaysia; 2 Department of Land Management, Faculty of Agriculture, Universiti Putra Malaysia, Serdang, Selangor, Malaysia; 3 Veterinary Research Institute, Ipoh, Perak, Malaysia; 4 Department of Veterinary Public Health and Preventive Medicine, Faculty of Veterinary Medicine, University of Maiduguri, Borno State, Nigeria; J Craig Venter Institute, UNITED STATES

## Abstract

Soil is considered to be a major reservoir of *Burkholderia pseudomallei* in the environment. This paper investigates soil physicochemical properties that may influence presence of *B*. *pseudomallei* in soil samples from small ruminant farms in Peninsular Malaysia. Soil samples were collected from the farms and cultured for *B*. *pseudomallei*. The texture, organic matter and water contents, pH, elemental contents, cation exchange capacities, carbon, sulfur and nitrogen contents were determined. Analysis of soil samples that were positive and negative for *B*. *pseudomallei* using multivariable logistic regression found that the odds of bacterial isolation from soil was significantly higher for samples with higher contents of iron (OR = 1.01, 95%CI = 1.00–1.02, p = 0.03), water (OR = 1.28, 95%CI = 1.05–1.55, p = 0.01) and clay (OR = 1.54, 95%CI = 1.15–2.06, p = 0.004) compared to the odds of isolation in samples with lower contents of the above variables. These three factors may have favored the survival of *B*. *pseudomallei* because iron regulates expression of respiratory enzymes, while water is essential for soil ecology and agent’s biological processes and clay retains water and nutrients.

## Introduction

Soil is considered to be a major reservoir of *Bukholderia pseudomallei*, the causative agent of melioidosis in humans and animals. Factors responsible for the occurrence of *B*. *pseudomallei* in endemic areas are poorly understood, although studies have suggested several properties related to soil that may influence the distribution of the organism [1, 2]. Physicochemical properties such as temperature, pH, soil water contents and sunlight have been shown to influence the survival of *B*. *pseudomallei* in soil under laboratory conditions [3, 4]. In addition, the intensity of rainfall, season, changes in landscape, soil type, farm management and human activities were suggested to influence the occurrence of melioidosis in endemic areas [5–10].

Defining the pattern of *B*. *pseudomallei* distribution may help in assessing the risk of melioidosis infection [11]. In Malaysia, the prevalence of the organism in soil is unknown however the overall seroprevalence of melioidosis in livestock was reported to be 5.7% with the reactor rates in sheep and goats found to be 13.6% and 2.6% respectively during a 10-year study period [12]. Sampling of soil from the endemic region of Australia found a prevalence of 14% [13]. Contact with contaminated environmental reservoirs appeared to be an important risk factor for infection among animals. Soil movement, heavy rainfall resulting in water run-off and/or aerosolization of *B*. *pseudomallei* during high velocity wind were suggested to be risk factors for infection [14]. The presence of some basic cations, macro and microelements, organic matter and the cation exchange capacity of soil were found to be potential properties of soil that could influence microbial growth in general [15–18]. However, information on the effects of these substances on the presence of *B*. *pseudomallei* in soils of the endemic areas is limited [19, 20]. This study investigates the physicochemical properties of soil that may influence the presence of *B*. *pseudomallei* in small ruminant farms in Malaysia.

## Materials and Methods

### Study area

Malaysia is a country located in the Southeast Asian region and is comprised of two main parts, the West Malaysia (Peninsular Malaysia) and East Malaysia (Sabah and Sarawak on the Borneo Island). The Peninsular Malaysia is made up of 11 states and two Federal territories (Wilayah Persekutuan and Putrajaya) while the East is comprised of Sabah and Sarawak states and the Federal territory of Labuan all located on the Borneo Island. The two parts of Malaysia are separated by the South China Sea [21]. Peninsular Malaysia covers an area of 131,598 square kilometers sharing common borders with Thailand in the north and Singapore in the south. The 11 states in Peninsular Malaysia include Johor, Kedah, Kelantan, Melaka, Negeri Sembilan, Pahang, Perak, Perlis, Pulau Pinang, Selangor and Terengganu [22]. The country lies entirely in the equatorial zone and is situated in the northern latitude between 1° and 6° N and the eastern longitude from 100° to 103°E. The climate of Peninsular Malaysia is influenced by two monsoon seasons, the northeast monsoon, which starts in November and ends in March and the southwest monsoon, which starts in May and ends in September. The northeast monsoon is usually characterized by prolonged heavy rainfall in northern and eastern regions of the Peninsular Malaysia causing severe floods in low-lying areas especially the east coast states of Kelantan, Terengganu, Pahang and east Johor. On the other hand, the southwest monsoon is characterized by drier conditions with less amount of rainfall throughout the Peninsular [22, 23]. According to the Malaysian Meteorological Department, Peninsular Malaysia has an average rainfall of 2,400 mm with hot and humid weather throughout the year.

### Study design and study population

Goat and sheep farms were selected from four states in Peninsular Malaysia and visited for sample collection. The states from which farms were selected included Negeri Sembilan, Pahang, Perak and Selangor states. Letters requesting for the farmers to participate in the study were sent to the individual farmers before commencement of the study. Only those that indicated their willingness and agreed to participate were visited. At each of these farms, the farmers were interviewed using a structured closed-ended questionnaire. The farmers gave written consents for the use of the questionnaire results. In fact, The questionnaire was designed to capture information on farm demography, farm size, small ruminants population size, breeds, farm management, animal health status, source of water and presence of water bodies around farm, soil conditions such as soil pH, applications of fertilizers, herbicides or lime and soil-related activities as well as occurrence of adverse climatic events such as drought, erosion, landslide, water logging etc. around farm.

### Sample collection and bacterial isolation

A total of 60 small ruminants farms were selected from a database of farms utilized for disease surveillance and monitoring by the Department of Veterinary Services (DVS) and the Veterinary Research Institute (VRI) Ipoh, Malaysia. A detailed description of the database was published by Musa et al., [10]. The farms visited were categorized based on management practice as intensive (21), semi-intensive (30) and extensive (9) farms. For the intensively managed farms, samples were taken within the farm premises especially near the sources of the animals’ feed and water. In farms practicing semi-intensive and extensive systems, soil samples were taken within the farm premise near sources of animals’ feed and water and from areas where the animals graze. The soil sampling procedure was performed as described by Chantratita *et al*., [24]. About 500ߞ700g of soil was taken at depth of 30cm from the surface. Triplicate soil samples were collected from three different spots. One of the sampling spots in each farm was located near the animals’ source of food one from their source of water and the third at least 100m away from the first two and roughly formed the third point of a triangle marked on the ground. A total of 180 soil samples (three from each farm) were collected for this work. Samples were sealed, labeled and transported to laboratory for processing. The sampling utensils were cleaned and disinfected between each use.

The soil samples were processed according to the standard method procedure by Limmathurotsakul et al [25] with slight modification. Briefly, 100g of soil sample was mixed with 100ml of distilled water, shaken for 1minute and allowed to sediment overnight. Then 100μl of supernatant was plated on Ashdown‘s agar, incubated at 37°C and observed for growth. Additionally, an enrichment process where 1ml of the supernatant was added to 9ml of threonine basal salt solution supplemented with 50mg/l polymixin B as enrichment broth was carried out. Both broths and agar plates were incubated at 37°C and observed for 48 hours. Suspected isolates were screened using catalase and oxidase tests, colony appearance and growth on Ashdown’s agar and presumptive isolates were stored in glycerol/ brain heart infusion (BHI) medium at -20°C until confirmation using PCR as described by Brook et al., [26].

### Identification of *Burkholderia pseudomallei*

Suspected colonies of culturable *B*. *pseudomallei* were initially screened on the bases of colony appearance, growth on Ashdown’s agar, catalase and oxidase tests. The characteristic colony morphological descriptions as purple, flat, dry and wrinkled by Chantrantita et al. [27] were utilized as guide in the screening. Colonies of suspected to be *B*. *pseudomallei* were further screened using API 20NE kits according to manufacturer’s instructions. Presumptive isolates that were positive using the API 20NE were confirmed using polymerase chain reaction (PCR) amplification. The PCR protocol was as described by Brook et al., [26] using PPM3 and PPM4 primers selected from the 16S rRNA region of *B*. *pseudomallei*. The sequences were from position 452 to 472 (PPM3-forward primer) 5' AATCATTCTGGCT AATACCCG 3' and position 1023 to 1042 (PPM4-reverse primer) 5' CGGTTCTCTTTCGAGCTCG 3'. The DNA templates were prepared from the suspected isolates using boiling method. The positive control was a DNA of previously confirmed *B*. *pseudomallei* isolated from kidney of rabbit, which died of melioidosis. The negative control used was sterile distilled water. The amplification process was carried out using a thermalcycler (MyCyler^®^, Bio Rad, US). The PCR amplification consisted of 30 cycles of 1 minute at 94°C, 30 seconds at 54°C and 2 minutes at 72°C, with a final extension step of 10 minutes at 72°C. Products were visualized by electrophoresis on a 1.0% agarose gel stained with 0.1% ethidium bromide.

### Determination of physicochemical properties of samples

Before analyses was performed to determine the soil physicochemical properties, soil samples were divided into two groups (based on the PCR finding); samples positive for *B*. *pseudomallei* (n = 32) and samples negative for *B*. *pseudomallei* (n = 28). Samples from each farm were pooled by mixing 200g from two of the triplicate samples obtained from the farm. Determination of water contents was carried out according the procedure described by Forster [28]. Briefly, this involved measuring the weights of moist soil samples after which the samples were dried and re-weighed to obtain the percentage weight loss. Each sample from the two groups was separately air-dried, ground and sieved. Soil textures were analyzed using mechanical method as described by Miller and Miller [29]. Organic matter contents were determined using the loss on ignition (LOI) method as described by Salehi et al., [30] where samples were dried in the oven at 105°C overnight then cooled in a desiccator. Then 200mg of each of the samples were weighed and combusted at 550°C for 2hours in a muffle furnace (Model 1400 Furnace, Humboldt, Germany). After combustion, the samples were cooled in a desiccator and weighed again. An estimation of soil organic matter percentage was calculated as the difference between the oven-dried weights and the combusted weights. The pH values were measured at room temperature using a pH meter after samples were dissolved in equal amounts of distilled water. The trace elements that included iron, copper, manganese and zinc as well as basic cations that included sodium, magnesium, calcium and potassium were determined using atomic absorption spectrophotometer (PerkinElmer, Inc., Shelton, CT, USA) according to the manufacturer’s instructions and published guides for soil samples [28, 31, 32]. The cation exchange capacities (CEC), measured in CentMol/l (CMol-^1^l) of samples were determined using auto-analyzer (QuickChem^®^ 8000 Series, Flow Injection Analysis System, Lachat Instruments, Loveland, USA) in accordance with the manufacturer’s guide. Carbon, nitrogen and sulfur contents were analyzed using CNS elemental analyzer (TruMac CNS Analyzer, Leco, US) according to the manufacturer’s instruction.

### Data Analysis

Data were entered into Microsoft Excel and analyzed using JMP^®^ for Mac (version 9.0.1 SAS Institute inc., Cary, NC, USA). Mean values of physicochemical properties were compared between *B*. *pseudomallei* positive and negative samples using independent T-tests with p-values <0.05 considered to be significant. A Chi-square test for association was performed to examine relationship between presence of *B*. *pseudomallei* in soil and farm management type and the type small ruminant kept in the farm. A multivariable logistic regression analysis was carried out using backward stepwise method in which soil parameter with univariable level of significance p<0.25 were selected for inclusion in the base model, and variables were excluded if the p-value was >0.05 and did not meaningfully alter the point estimates of the remaining variables. Exploratory correlation analysis shows significant collinearity between the predictor variables namely CEC, organic matter, carbon and nitrogen contents and so were excluded from the analysis one at a time. The outcome variable used in the analysis was the outcome of PCR confirmation of *B*. *pseudomallei* (positive/negative) isolation from soil sample. Chi-square test for independence was used to assess association between the independent variables. The overall goodness-of-fit of the model to the data was examined using the Hosmer-Lemeshow test.

## Results

### Bacterial isolation

Table 1 (S1 Table) shows the results of *B*. *pseudomallei* isolation from soil samples collected from small ruminant farms from the four states in this study. Screening of the suspected *B*. *pseudomallei* isolates using the API 20NE kits found samples from 33 farms to be positive while those from 27 farms were negative. However, PCR amplifications of these samples confirmed 32 were positive while 28 negative. One sample each from Pahang and Selangor, which initially gave negative results using the API 20NE kit yielded positive results using PCR amplification. In the same vein, a sample from Negeri Sembilan and two samples from Perak that were positive using the API 20NE kits was negative using PCR amplification. The results from the confirmatory PCR were used for further analysis of the data in this study.

**Table 1:** Results of *Burkholderia pseudomallei* isolates screening using API 20NE kits and PCR confirmation from soil samples obtained from small ruminant farms in Peninsular Malaysia.

State	API 20NE			PCR		
	Positive (%) n = 33	Negative (%) n = 27	Subtotal n = 60	Positive (%) n = 32	Negative (%) n = 28	Subtotal n = 60
N. Sembilan	7 (63.6)	4 (36.4)	11(100)	6(54.5)	5(45.5)	11(100)
Pahang	11(55.0)	9(45.0)	20 (100)	12 (60.0)	8 (40.0)	20 (100)
Perak	11(61.1)	7 (38.9.)	18 (100)	9(50.0)	9(50.0)	18 (100)
Selangor	4 (36.4)	7 (63.6)	11(100)	5(45.5)	6 (54.5)	11 (100)

We found no significant association between the proportions of isolation of *B*. *pseudomallei* from soil samples to the type of management systems (intensive, semi-intensive and extensive) and the type of animals kept in the farm (goats only, sheep only and mixed) *(χ*^*2*^ = 2.41, df = 2, p = 0.29 and *χ*^*2*^ = 0.10, df = 2, p = 0.94, respectively).

### Univariable analysis of physicochemical parameters of soil samples

The descriptive statistics and mean percentages of the physicochemical parameters of *B*. *pseudomallei* positive and negative soil samples from the study farms are presented in Table 2 (S1 Table). In terms of soil texture, there were significant differences (p<0.05) between the means of clay and silt contents of *B*. *pseudomallei* positive compared to those of the negative samples. Similarly, comparisons of the means of organic matter, water, clay, iron, carbon and nitrogen contents as well as the cation exchange capacities between *B*. *pseudomallei* -negative and positive soil samples using T-test showed significant differences (p<0.05) between the two groups.

**Table 2:** Descriptive statistics and mean percentages of the physicochemical variables of *B*. *pseudomallei* positive and negative soil samples from small ruminant farms in Malaysia.

Variable	Descriptive			Positive (n = 32)		Negative (n = 28)		p-value
	Range	Mean	SD	Mean	SEM	Mean	SEM	
% Sand	90.80	39.05	22.06	33.75	3.57	45.12	4.33	0.047*
% Clay	56.10	7.42	7.25	9.43	1.61	5.13	0.58	0.016*
% Silt	88.60	53.48	21.53	57.34	3.83	49.07	3.95	0.137
% O.M.	0.63–9.07	1.97	1.24	2.49	0.26	1.38	0.10	0.002*
% Water	2.23–18.36	9.74	4.39	11.08	0.74	8.24	0.80	0.011*
Soil pH	2.49–5.70	3.92	0.89	3.78	0.16	4.09	0.16	0.192
CEC (CMol/l)	1.55–269.0	88.24	48.60	105.86	9.39	68.11	6.36	0.002*
Fe (mg/l)	8.53–819.2	177.89	199.09	247.00	42.08	98.91	18.36	0.002*
Cu (mg/l)	0.26–6.23	0.67	0.86	0.45	0.05	0.92	0.22	0.051
Zn (mg/l)	0.45–3.21	0.82	0.49	0.82	0.08	0.81	0.10	0.945
Mn (mg/l)	0.11–4.28	0.82	0.81	0.86	0.17	0.76	0.12	0.623
Na (mg/l)	0.20–20.0	1.15	2.74	0.86	0.61	0.76	0.32	0.676
Mg (mg/l)	0.02–0.52	0.12	0.14	0.12	0.03	0.11	0.02	0.666
Ca (mg/l)	8x10^-3^–10.36	1.42	2.02	1.55	0.35	1.27	0.39	0.589
K (mg/l)	0.50–52.78	10.06	11.94	9.58	1.76	10.60	2.65	0.748
% Carbon	0.16–4.85	0.90	0.69	2.49	0.14	1.38	0.05	0.002*
% Nitrogen	3x10^-3^–0.40	0.85	0.55	0.099	0.011	0.068	0.006	0.025*
% Sulfur	7x10^-4^–0.098	0.011	0.16	0.011	0.002	0.010	0.003	0.838
C/N ratio	2.24–110.60	14.46	19.60	17.05	4.18	11.51	2.55	0.26

SD = standard deviation, SEM = standard error of mean, %=percentage, O.M. = Organic matter, CEC = cation exchange capacity, C/N = carbon/nitrogen.

*There was a significant different between the means at p<0.05.

### Multivariable Logistic Regression

The multivariable logistic regression of the soil properties influencing the presence of *B*. *pseudomallei* in soil samples is presented in Table 3. The Hosmer-Lemeshow goodness of fit test showed that the model significantly fitted the data (χ^*2*^ = 3.67, df = 8, p = 0.88). The final model revealed that only three of the factors tested remained significant (p<0.05) as predictors of the presence of *B*. *pseudomallei* in the soil samples when the effects of other variables were accounted for.

**Table 3:** Multivariable logistic regression of soil properties associated with the presence of *B*. *pseudomallei* in soils from small ruminant farms in Peninsular Malaysia.

Variable	Positive (n = 32) Mean±SEM	Negative (n = 28) Mean±SEM	Odds Ratio	OR 95%CI	P-value
Iron contents	247.00±42.08	98.91±18.36	1.01	1.00–1.02	0.034*
Copper contents	0.45±0.05	0.92±0.22	0.14	0.02–1.30	0.083
Soil pH	3.78±0.16	4.09±0.16	0.04	0.15–1.01	0.051
% Clay contents	9.43±1.61	5.13±0.58	1.54	1.15–2.06	0.004*
% Water contents	11.08±0.74	8.24±0.80	1.28	1.05–1.55	0.013*

SEM = standard error of mean, CI = confidence interval, %=percentage.

* = significantly different

We found that when compared with *B*. *pseudomallei*-negative soil sample, a positive soil sample was significantly more likely to have higher: 1. iron content, whereby the odds of isolating *B*. *pseudomallei* from soil sample will increase by a factor multiplied by 1.01 for every unit increase the soil iron content; 2. water content, whereby the odds of isolating *B*. *pseudomallei* from soil sample will increase by a factor multiplied by 1.28 for every percentage increase in soil water content, 3. clay content whereby odds of isolating *B*. *pseudomallei* from soil sample will increase by a factor multiplied by 1.54 for every unit increase in clay content of the soil.

Other soil physicochemical properties included in the final model were soil pH and its copper contents that were not significant but were considered biologically meaningful and important in contributing to the explanation of the variation seen in the study.

## Discussion

Soil texture, the relative proportion of sand, silt and clay contents of the soil, have been suggested to be essential for understanding the physical, physicochemical and biological properties of soil [33]. In this study, we screened several physicochemical properties using the univariable analysis and identified seven that significantly influenced the presence of *B*. *pseudomallei*. However, when these variables were considered together in a multivariable logistic regression, only five remained in the final model with three associations found to be significant while the other two were approaching significance. We believe that the power of the study may not be adequate to detect small differences in association of the latter two variables because our sample size was not large. It is also possible that the method used to detect the presence of the bacteria in this study (ie culture and identification) prior to PCR is less sensitive than direct PCR amplication from soil samples [34]..

Our finding on the increased likelihood of isolating *B*. *pseudomallei* when soil has high contents of clay agrees with report from previous researches in the endemic regions of Australia [1, 35, 36]. Clay soil tends to support survival of *B*. *pseudomallei* because clay has excellent water and nutrient retention capabilities due to its large surface area and chemical activity [7]. Our finding also agrees with other studies reporting isolation of *B*. *pseudomallei* from rice fields [37, 38] that are predominantly clayey in texture and have the tendency for waterlogging [39]. In general, clay is known to support bacteria due to its small pore size, enabling this structure to withhold nutrients and water. It has also been reported that electrostatic interactions of clay particles with bacterial extracellular polysaccharides [17] popularly known as “clay hutches” [40] aid the survival of *B*. *pseudomallei*. Clay contents of soils in general have been reported to influence other soil parameters such as the “protected biomass” availability of substrates for activity of microorganisms present in soil, soil porosity and ecology and microenvironment of soil microorganisms [41].

The soil sand content was not significant in our final model. Reports have been inconsistent on the findings about this variable as some study have found that soil with high sand contents is less likely to support the persistence of *B*. *pseudomallei* due to low water and nutrient contents [42] while others have recorded that, *B*. *pseudomallei* has been isolated from soil samples with high percentage sand contents and poor nutrient contents [13]. We believe that complex interaction exists between parameters in the soil beyond the soil texture alone and more work need to be done to arrive at conclusion about the organism’s niche preferences for perpetuation.

Of the four trace elements that included iron (Fe), copper (Cu), zinc (Zn) and manganese (Mn) investigated in the study, only Fe contents was found to be significant, consistent with the recent finding published by Wang-ngarm et al. [43]. Iron in soil exists in ferrous (Fe^2+^) and ferric (Fe^3+^) forms where the ferrous being forms available for plant and microbial activity [44, 45]. The availability of the iron in these forms is in turn affected by other soil properties such as pH, aeration and organic matter contents and plants adaptation. The iron oxides and hydroxides in soil are responsible for the yellowish and reddish color in soil [44, 45]. These colors reflect the high iron contents which has been found to be associated with the occurrence of *B*. *pseudomallei* [36]. Our finding is supported by Draper et al. [46] who found strong association between occurrence of *B*. *pseudomallei* in water with higher iron contents. In addition, laboratory study where iron (in form FeSO_4_) was added to the growth medium of *B*. *pseudomallei* showed an increased biofilm formation which may lead to improved resilience of *B*. *pseudomallei* against environmental inhibitory factors [47]. In the same vein, increased levels of intracellular iron has been shown to enhance the invasion efficiency, replication and intracellular survival of this organism [48]. Furthermore, iron (Ferric ion) regulate the expression of respiratory enzymes involved in *B*. *pseudomallei*’s biological processes [49, 50] therefore enhancing their survival. The acquisition of these iron was suggested to be regulated by siderophore biosynthesis in *B*. *pseudomallei* [50]. Successful competition for iron has been reported to be an essential aspect of *B*. *pseudomallei* ability to establish infection in a host [51]. Iron is also utilized by the agent to form superoxide, FeSOD (iron superoxide dismutase) which protects it against the “host” immune system [52] thereby enhancing its survivability in host’s system.

Our findings on iron contradicts those of Baker et al. [13] who reported that the iron contents of soil samples positive for *B*. *pseudomallei* were significantly lower when compared with samples that were negative. This inconsistency may be due to the way data were analysed. In our study, data were analyzed using a multivariable logistic regression, which consider putative variables that may influence the presence of *B*. *pseudomallei* together in a model, while the data from Baker et al. were analyzed using univariable analysis, which consider the variables individually thus may not have accounted for the effects of other variables in the study. Alternatively, the disparity may also be due to the presence of other unexplained interacting factors that might not have been adequately captured in our study. Complex interactions amongst several factors have been suggested to be the major determinants of *B*. *pseudomallei* and melioidosis in the endemic regions [8, 39, 53].

Copper exists in soil in silicate minerals or carbonates forms which are largely unavailable for plant and microbial activities. The form available is cation (Cu^2+^) found usually on surfaces of clay minerals or in association with organic matter where the availability is in turn influenced by soil pH and organic matter contents [54].We found a significant difference between copper contents of samples that were negative for *B*. *pseudomallei* to those positive samples in the univariable analysis, consistent with that found by Baker et al. [13]. However the variable did not remain significant when other variables were accounted for. The information on the exact role played by soil copper contents on survivability of *B*. *pseudomallei* in the environment could not be obtained from literature to fully understand the effects of copper on the agent. However, copper has been found to slow down biofilm formation in other Gram-negative bacteria such as *Legionella pneumophila* in water due to its toxic or inhibitory effect on bacteria [55, 56].

Our finding that water contents in samples affects the likelihood of the organism’s survival in those soil samples is expected as water is essential in the maintenance of ecological balance in soil microorganisms, as availability of nutrient and the integrity of bacterial membranes are dependent on the soil solution which in turn depends of soil water [57]. Water in soil is maintained in network of pores where gravitational water circulates in macropores and capillary water in the micropores [58]. Consequently, the water in micropore offers favorable environment for bacteria by protecting them from desiccation and from exogenous toxic water-soluble substances. To highlight the importance of soil water contents on the survival of *B*. *pseudomallei*, a laboratory investigations by Tong et al [3] and by Chen et al [4] have both shown that water in combination with pH and temperature were the three major ecological factors affecting survival of *B*. *pseudomallei* in soil. Pumpuang et al., [59] showed that the organism can survive in distilled water for more than 16 years [59] which underscore the importance of water in survival and maintenance of this organism even in the absence of other nutrients. The mean percentage water contents of the positive soil samples in our study was about 11% and this is observed to be well within the range of positive *B*. *psseudomallei* sites in Thailand with 9–18% moisture contents [60]. However, *B*. *pseudomallei* may survive even in the environment with lower moisture than those aforementioned such as the desert [61].

Soil pH was included in the logistic regression model as one of the predictors for the occurrence of *B*. *pseudomallei* in soil even though the association was not significant. Laboratory investigations by Tong et al [3] and another by Chen et al [4] have both shown that pH, together with water and temperature were the three major ecological factors affecting survival of *B*. *pseudomallei* in soil under laboratory conditions. Under field conditions, lower pH has been found in other endemic regions such Thailand [60] and Australia [7] to promote the presence of *B*. *pseudomallei*,. The bacteria has been observed to prefer more acidic medium [62] for its growth and survival and according to Dejsirilert et al., [63], acidic medium probably enhanced the production of acid phosphatase in the bacteria which in turn aid its survival in the environment. However, earlier study in soils from Malaysia isolated *B*. *pseudomallei* from soils of varying pH values between 2.8 and 7.4 [64] indicating the organism’s preference for more acidic to neutral medium. Our previous findings on the decreasing risk of melioidosis in farms that have treated their soil with lime further support this finding [10].

In conclusion, this study found that iron, water and clay contents of soil samples were the three most important factors influencing the occurrence of *B*. *pseudomallei* in farm environment in Peninsular Malaysia. Higher iron, clay and water contents of soil samples appear to support the presence of *B*. *pseudomallei* in the soil. The copper contents of soil also appear to meaningfully decrease the likelihood of the pathogen survival. This study revealed information on the physicochemical properties of soil that may influence the occurrence of *B*. *pseudomallei* in soil in Malaysia. However, the sample size available for this study is limited therefore may not have uncovered other factors that could have played significant role in the survival of *B*. *pseudomallei* in the farm environment.

## Supporting Information

S1 Table Physicochemical Properties of Soil in Small Ruminant Farms. (XLSX)
